# Interdisciplinary Approaches to Sports in the 21st Century

**DOI:** 10.3390/sports14100448

**Published:** 2026-10-10

**Authors:** Paweł Adam Piepiora, Zbigniew Norbert Piepiora

**Affiliations:** 1 Faculty of Physical Education and Sports, Wroclaw University of Health and Sport Sciences, 51-612 Wrocław, Poland; 2 Faculty of Environmental Engineering and Geodesy, Wrocław University of Environmental and Life Sciences, 50-357 Wrocław, Poland

## 1. Introduction

Sports is an important area of modern social life [1]. The struggles of athletes gather crowds of fans, as new records are not infrequently set [2]. These records are achieved through the comprehensive preparation of athletes, which considers new discoveries from various scientific disciplines [3]. Sport activities in any form are becoming increasingly popular [4]. Sport is also an attractive way to spend free time [5]. Sport provides psychophysical benefits to athletes through training, competing, restoring, and actively exploring the world [6].

In the 21st century, sports have ceased to be merely the domain of motor skills, intuition, and talent, backed by systematic training. Contemporary high-level athletics is also based on the latest scientific advances in various fields. Therefore, the results achieved are no longer solely the accomplishments of athletes and their coaches. Instead, athletes are supported by multidisciplinary teams of specialists. In light of this, the goal of this research topic, “Interdisciplinary Approaches to Sports in the 21st Century”, is to present current discoveries that may help athletes achieve ever-better athletic results. In this collection, we present fourteen works by authors who have chosen to publish their achievements.

## 2. Overview of Published Articles

Liu et al. (contribution 1) examined helmsperson leadership in terms of the temporal dynamics of management characterized by high responsibility and low visibility. The authors expanded the theoretical boundaries of relational leadership by extending the concept of relationality from interpersonal interactions to the leader’s relationships with the system, risk, and the environment. The authors introduced the “visibility-accountability” analytical framework into leadership theory, revealing its fundamental characteristics and mode of operation.

Jin et al. (contribution 2) studied motor–cognitive associations in older adults. The researchers analyzed the inter-relationships between motor coordination abilities and cognitive functions in older adults, with the aim of developing a preliminary diagnostic tool that facilitates the early detection of motor–cognitive decline. The authors constructed a tool that helps individuals monitor their motor and cognitive health.

Pires et al. (contribution 3) examined the associations between psychological coping skills and player behaviors during transition moments in male youth football. This exploratory pilot study aimed to develop a diagnostic framework that links individual behaviors during football attack–defense transition moments with psychological attributes.

de Miranda Rohlfs et al. (contribution 4) studied mood states, injury status, and countermovement jump performance in high-level Brazilian athletes. The authors found that negative mood profiles, particularly those characterized by elevated levels of anger and fatigue, are associated with increased risk of injury. Mood assessment results were not associated with variability in performance on the counter-movement jump with arm swing.

Assadourian et al. (contribution 5) studied neuropsychological performance in university-level football athletes. The researchers found significant negative correlations between quantitative electroencephalography profiles associated with burnout, ADHD, depression, and anxiety and specific subcomponents of attention. This finding suggests a link between brain activity related to mental health and attentional performance.

Zhu et al. (contribution 6) conducted a systematic review and meta-analysis of the effects of perceptual-cognitive training on anticipation and decision-making skills in team sports. The researchers found that the effects of training interventions vary depending on how stimuli are presented and the duration of the intervention. The researchers concluded that perceptual-cognitive training improves performance on the field, but its transfer effects are limited. Therefore, future interventions should use virtual reality to present training stimuli and consider sport-specific reactions of participants so that interventions reflect real-world game scenarios.

Lawrence and Sorra (contribution 7) critically analyzed low-level laser therapy for acute tissue injury or sports performance recovery. The researchers concluded that the variations in the parameters of low-level laser therapy administration and in study results underscore the need to develop clear treatment guidelines for physical therapists, medical doctors, coaches, and athletes.

Lu (contribution 8) examined the motivations for and strategies of sport-based corporate social responsibility using multiple case studies of Taiwanese enterprises as examples. Through in-depth interviews and numerous case studies, the author analyzed the common features and unique characteristics of sports-based corporate social responsibility. A detailed picture is presented of the current state of affairs and the challenges facing companies engaged in sports-based corporate social responsibility and sustainable development.

Plakias et al. (contribution 9) studied tactical situations and playing styles as key performance indicators in soccer. The researchers found that a possession-based style of play, counterattacks during offensive transitions, and a balanced, aggressive defensive strategy significantly increase a team’s chances of winning. Furthermore, successful teams typically focus on attacks through the center of the field, minimize crosses, and execute strategic plays that lead to shots on goal with minimal ball possession time. Consequently, the use of specific tactical approaches significantly impacts soccer match outcomes, underscoring the importance of considering tactical aspects as key performance indicators.

Codonhato et al. (contribution 10) examined the psychometric properties of the Performance Enhancement Attitude Scale for Brazilian athletes. The results provided satisfactory evidence of validity based on the response process, internal structure, and reliability. Thus, the Performance Enhancement Attitude Scale questionnaire was adopted for use with Brazilian athletes.

Santos et al. (contribution 11) conducted an exploratory study of women’s experiences in Portuguese soccer organizations. The authors found that the soccer community emphasizes sex differences, with women being more visible, which leads to them being assigned stereotypical roles associated with traditional femininity. Consequently, women conform to the norms imposed by the dominant group—men—and accept the limitation of their activities to the spaces assigned to them by hegemonic gender ideologies and practices.

Hwang et al. (contribution 12) studied climate change awareness and pro-environmental intentions among sports fans. The authors employed an extended model of the theory of planned behavior that incorporates climate change awareness. The researchers found that climate change awareness positively predicts the intention to reduce the use of single-use plastics in the context of sports fandom.

Oh et al. (contribution 13) compared audience behavior between eSports and traditional sports fans, finding differences, but the decisive factor affecting this difference was whether or not they had experience playing sports, rather than the content.

Finally, Zhu et al. (contribution 14) examined visual search behavior in volleyball spiking defense from different viewing perspectives. The researchers found that competitive and semi-elite players were more accurate than beginners. Elite players achieved better results, and the distribution of visual attention across areas of interest was similar among groups of participants. Furthermore, from the end-line perspective, the participants primarily relied on global perception and peripheral vision, focusing more on the playmaker’s zone or the attacker’s torso. In contrast, from the field perspective, participants used more fixations, focusing more intensely on the attacker’s specific movements.

## 3. Conclusions

This collection of works dedicated to contemporary interdisciplinary approaches to sports comprises a broad spectrum of research that highlights the relevance of the topics addressed. A strong integration of sports science, neurobiology, psychology, and management is evident, centered around optimizing human potential, psychophysical health, and understanding social behavior, as also reflected in the methodologies applied in individual studies.

These findings confirm that optimizing athletic performance and sports management require a seamless integration of knowledge from multiple previously distinct fields. Therefore, contemporary sports research necessitates abandoning narrow specializations, as competitive success relies on multifaceted approaches to sports.

Consequently, an interdisciplinary approach to sports in the 21st century demands a shift away from intuition toward data-driven implementations while charting new avenues for scientific inquiry. The future of athletic progress hinges on the proficiency with which researchers and practitioners directly translate these interdisciplinary insights from laboratories to fields, stadiums, and coaching offices.
